# A Low Dose of Naloxone Added to Ropivacaine Prolongs Femoral Nerve Blockade: A Randomized Clinical Trial

**DOI:** 10.1155/2021/6639009

**Published:** 2021-01-31

**Authors:** Seung Cheol Lee, Jeong Ho Kim, So Ron Choi, Sang Yoong Park

**Affiliations:** Department of Anesthesiology and Pain Medicine, Dong-A University College of Medicine, Busan 49201, Republic of Korea

## Abstract

Femoral nerve blocks (FNBs) are used as safe and useful procedures to control severe postoperative pain from total knee arthroplasty (TKA). Various adjuvants have been used to prolong the duration of the local anesthetic blockade. This study evaluated whether a low dose of naloxone administered with local anesthetics prolongs the duration of FNB. A prospective, randomized double-blind controlled study was conducted with 74 patients undergoing unilateral TKA. Through a single-bolus administration guided by ultrasound, the control group (group C) received 20 mL of 0.375% ropivacaine, while the naloxone group (group N) received 20 mL of 0.375% ropivacaine with 100 ng of naloxone. The time elapsed before the first analgesia request, the total amount of opioids consumed at 24 h postoperatively, the onset time of the sensory blockade, the visual analog pain scale (VAS) scores after arriving at the recovery room, after 6, 12, 18, and 24 h at rest and after 12, 18, and 24 h of activity, the quadricep strength before the FNB procedure and at 12 and 24 h postoperatively, the quality of sleep on the first night after surgery, the satisfaction score, and the incidence of postoperative complications were recorded. The time elapsed before the first analgesia request was significantly longer in group N (735.5 ± 187.2 min) than that in group C (602.6 ± 210.4 min) (*P*=0.003). The total dose of supplementary opioids consumed at 24 h postoperatively was significantly lower in group N (312.4 ± 141.7 *μ*g) than that in group C (456.5 ± 279.5 *μ*g) (*P*=0.007). Lower VAS scores were recorded in group N than that in group C at rest and during knee activity (rest, 12 h, *P*=0.001, 18 h, *P*=0.043; activity, 12 h, *P*=0.001). The addition of a low dose of naloxone to ropivacaine for FNB significantly delayed the first request for rescue analgesia and decreased the opioid consumption within 24 h, without significant complications.

## 1. Introduction

Severe pain always follows total knee arthroplasty (TKA) [1, 2]. The control of postoperative pain has been an essential step in facilitating a quick and functional recovery, which eventually yields patient satisfaction and an early return to productive society after TKA [3]. Opioids used for pain control have many side effects; consequently, different regional techniques, such as epidural analgesia, femoral nerve block (FNB), adductor canal block (ACB), peripheral nerve block, and local infiltration analgesia (LIA), have been investigated [1, 2, 4–6]. Among them, FNB, which can be implemented on the target side, is often selected and performed. Compared to ACB, FNB has no statistically significant differences in analgesic effects, quadricep strength, or functional recovery postoperatively [7]. While ACB is motor-sparing, it also appears to be partially sensory-sparing, as confirmed by anatomic facts as well as a previous study [8].

Naloxone is a known antagonist that is used to counter the effects of opioid overdose. Based on accumulating evidence, excitatory effects of opioids are selectively blocked by naloxone [9, 10]. This drug has been administered in low doses as an additive to local anesthetic (LA) medications to prolong motor and sensory nerve blocks during supraclavicular and axillary brachial plexus blockade and intrapleural infiltration [11–14].

A randomized double-blinded control study comparing the addition of a low dose of naloxone to ropivacaine with ropivacaine alone for FNB after TKA has not been conducted. Therefore, the present study tested the hypothesis that naloxone would prolong the duration of FNB and decrease the requirement for supplementary opioids for postoperative pain control.

## 2. Materials and Methods

This randomized, double-blinded, controlled clinical trial was conducted between January and July 2020. It was approved by the Research Ethics Committee of Dong-A University Hospital (reference number: DAUHIRB-19-199, October 09/2019) and registered with the Korean Clinical Trials (KCT0004632-January 16/2020-cris.nih.go.kr).

All patients were informed about the study and provided written consent. The inclusion criterion included primary unilateral TKA for osteoarthritis (OA) or rheumatoid arthritis (RA). The exclusion criteria included patients who refused to participate in the study or who had allergies or intolerance to one of the study drugs, pruritus, epilepsy, preexisting coagulopathy, mental illness, dementia, preexisting neuropathy on the operative leg, alcoholism, chronic opioid use (>3 months), a body mass index (BMI) > 35 or <20 kg/m^2^, age younger than 18 or older than 80 y, severe comorbidities, and American Society of Anesthesiologists (ASA) class 4 or 5. Upon admission to the hospital, the patients' demographics and perioperative data were recorded by a nurse who was a blinded observer.

Patients were then placed in a supine position with a cushion underneath their knee, resulting in a 45-degree angle at the knee. The quadriceps strength of both legs was assessed by placing the dynamometer on the anterior of the ankle between the malleoli. The patients were instructed to extend their legs three times each, with a 30 sec pause between each attempt, and the average was recorded. The patient's quadriceps strength grade was recorded in kilograms of force (kgf), as described by Bohannon [15]. After each attempt, patients rated their pain using the VAS. Range of motion (ROM) was assessed once each at 24 h preoperatively and postoperatively.

All patients received standardized analgesia upon hospitalization. Preoperative oral ibuprofen (200 mg, 3 times per day) was administered 2 days before surgery. All patients were randomly assigned to either the control group (group C) or the naloxone group (group N) via sequentially numbered (1–79), opaque-sealed envelope based on a computer-generated randomization list created by a blinded observer. Patients in group C received 20 mL of 0.375% ropivacaine with 1 mL of isotonic saline chloride (*n* = 37), and patients in group N received 20 mL of 0.375% ropivacaine with 1 mL (100 ng) of naloxone [16, 17] (*n* = 37). The patient and anesthesiologist were blinded to the group assignment. All local anesthetic solutions and naloxone were prepared by an independent researcher who was not involved in the performance of FNB, patient care, or data collection.

Before the premedication was administered, intravascular access was established in the operating room by inserting a peripheral venous cannula, and an infusion of 10 ml/kg lactated ringer solution was then administered. The premedication of all patients was performed by administering 0.1 mg/kg midazolam intravenously 20 min before the induction of anesthesia with an oxygen nasal cannula at a rate of 5 L/min. The patients were closely monitored using pulse oximetry, noninvasive blood pressure measurements, and three-lead electrocardiograms when they were admitted to the operating room.

A study assistant, not involved in the anesthesia of study participants, opened the sealed opaque envelope containing group allocations and prepared two 20 mL syringes of either 0.375% ropivacaine with 1 mL of naloxone or the same volume of isotonic saline chloride. Another assistant double-checked the procedure. Preparation of the study medicine was performed after enrollment and before block execution. Envelopes were resealed by the two study assistants. Envelopes remained sealed until completion of the study including data sampling and statistical analysis of outcomes. Thus, investigators, participants, care providers, outcome assessors, and statistician were all blinded to group allocations. Ultrasound-guided FNB (20 mL of 0.375% ropivacaine with or without 100 ng of naloxone, via a 22-gauge 2-inch Stimuplex A needle; B Braun Medical, Inc., Melsungen, Germany), was performed at the inguinal ligament level using a high-frequency linear ultrasound transducer (12–13 Hz; CX50 device; Philips Ultrasound, Bothell, WA, USA) with a nerve stimulator. After performing the block, the onset of the sensory block was assessed by pinprick using the jagged edges of a broken tongue depressor applied within the sensory distribution of the femoral nerves to the skin over the anterior and medial aspect of the thigh, medial aspect of the lower leg, and medial aspect of the knee to the great toe at an interval of 2 min to a maximum of 40 min. During this time, if all of the femoral nerve supply sensations were not blocked, then the outcome was considered as a failure to block.

Anesthesia was induced by intravenously injecting 2 mg/kg propofol and 0.9 *μ*g/kg fentanyl. Endotracheal intubation was facilitated by intravenously injecting 0.8 mg/kg rocuronium and was continued using 2% sevoflurane with medical oxygen to maintain a bispectral index of 35–60. Neuromuscular relaxation was maintained by intravenously injecting vecuronium top-ups of 0.05 mg/kg every hour. The lungs were mechanically ventilated to maintain the end-tidal CO_2_ pressure within 35 ± 5 mmHg. Additionally, a temperature probe was inserted. The operation was performed by a single experienced orthopedic surgeon (blinded to the study protocol) using the same technique in every patient.

Posterior capsule LIA (20 mL of 0.25% ropivacaine with 5 mg/L epinephrine was injected into the posterior capsule of the knee joint) was performed by an experienced orthopedic surgeon at the end of the operation. At the end of the surgery, residual muscle paralysis was reversed by intravenously injecting 4 mg/kg sugammadex, and the trachea was extubated when the extubation criteria were fulfilled.

After the operation, we administered acetaminophen (500 mg, twice a day) and ibuprofen (200 mg, 3 times in a day) to control postoperative pain. An intravenous patient-controlled analgesia (IVPCA) pump (Accufuser Plus M1015M, Woo Young Medical, Korea) was connected at the end of surgery to the patients whose VAS score at rest was greater than 4; the time of IVPCA initiation was recorded. The IVPCA was prepared at a total volume of 100 mL by mixing 20 *μ*g/kg fentanyl and 0.6 mg of ramosetron in normal saline. The baseline infusion rate, bolus demand dose, and lock-out time were 1 cc/h, 1 cc, and 10 min, respectively. The duration of postoperative analgesia was defined as the period between the end of LA solution and adjuvant injection for FNB and the postoperative initiation of IVPCA by the patient. Twenty-four hours after surgery, the remnant volume of IVPCA was checked, and the fentanyl dosage used up to that time point was calculated. After providing 1 h of postoperative care in the anesthesia recovery unit and ensuring that the patients were awake, all patients were returned to the inpatient unit.

All patients had been introduced to the VAS at their preoperative visit and had been taught how to use it to express their level of pain. The VAS score at rest was estimated immediately after arriving at the recovery room (0) and then at 6, 12, 18, and 24 h. The VAS score during activity of the knee joint was estimated at 12, 18, and 24 h.

The quadriceps strength of the operated leg was recorded before the FNB procedure and postoperatively at 12 and 24 h. The quality of sleep on the first night after surgery was measured using the Consensus Sleep Diary [18], and the quality of sleep was rated on a scale ranging from 0–4 points (4, very good; 3, good; 2, fair; 1, bad; and 0, very bad). Patient satisfaction scores of 0–100 were recorded. Any complications that occurred within 24 h were also recorded. Rescue antiemetics were administered according to the severity of nausea and vomiting. An intravenous injection of 4 mg of ondansetron was used to treat nausea and vomiting; if it was ineffective, 10 mg of intravenous metoclopramide was administered.

### 2.1. Sample Size Estimation

The sample size was calculated based on a previous study in which the patient received a similar postoperative analgesia regimen. In that study, the time of first rescue analgesia was 402 ± 54 min in the placebo group and 660 ± 48 min in the intervention group [14]. The study power was 90%, and the alpha level was 0.05. We needed to enroll 30 patients in each group to detect a 258 min difference in the time of first rescue analgesia between the two groups. Estimating a 20% dropout rate, we decided to recruit more than 36 patients for each group.

### 2.2. Statistical Analysis

Data are presented as the means (standard deviation (SD)), medians (interquartile range), or numbers of patients (%). We analyzed quantitative variables using Student's *t*-test or the Mann-Whitney *U* test, and qualitative variables were analyzed using the chi-square test or Fisher's exact test. All data were analyzed using SPSS® software, version 26.0 (SPSS, Inc., Chicago, IL, USA). A *P* value of <0.05 was considered statistically significant.

## 3. Results

**Figure 1:**
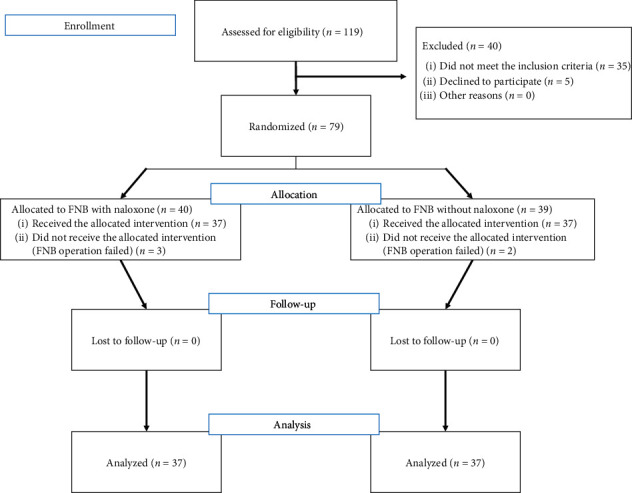
CONSORT diagram. Flow diagram of patients assessed using the protocol. FNB = femoral nerve block.

One hundred nineteen patients participated in the trial, but 45 were excluded for various reasons (Figure 1). The success rates for groups C and N were 94.9% and 92.5%, respectively. All 37 patients in each group completed the study protocol and were analyzed for the outcomes. The patients had similar demographics and operative data, as presented in Table 1.

Primary outcomes were the elapsed times before the patients first required analgesia and supplementary opioids. The elapsed time before the first request for analgesia was significantly longer in group N (735.5 ± 187.2 min) than that in group C (602.6 ± 210.4 min) (*P*=0.003). Additionally, a significantly lower total dose of supplementary opioids consumed was observed in group N (312.4 ± 141.7 *μ*g) than that in group C (456.5 ± 279.5 *μ*g) (*P*=0.007) (Table 2). A difference in the onset time of sensory blockade was not observed between the two groups (Table 2).

**Figure 2:**
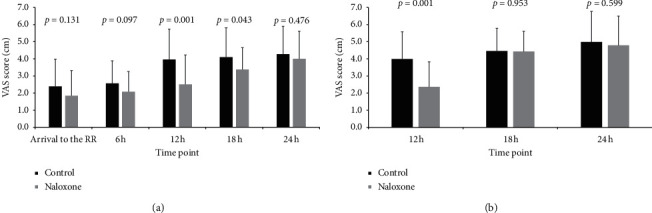
The VAS scores at rest (a) and during activity (b) of the knee at different time points before and after the operation. RR: recovery room; VAS: visual analog scale.

Lower VAS scores were recorded for group N than that for group C at rest and during activity of the knee (rest, 12 h, *P*=0.001, 18 h, *P*=0.043; activity, 12 h, *P*=0.001), but a statistically significant difference in the VAS scores was not observed between groups at 0, 6, and 24 h at rest and at 18 and 24 h during activity in the postoperative period (Figure 2).

**Figure 3:**
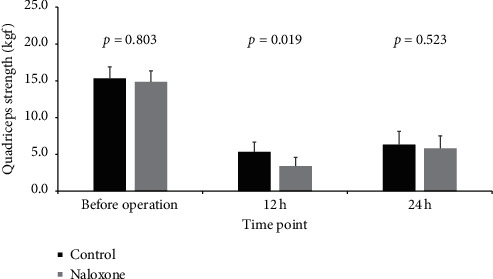
Strength of quadriceps in the operative knee at different time points before and after the operation. kgf: kilograms of force.

The quadricep strength of group C was superior to that of group N at 12 h postoperatively (*P*=0.019), but it was not inferior before the operation and at 24 h postoperatively (Figure 3).

The incidences of hypotension, nausea and vomiting, pruritus, and urinary retention were not significantly different between the two groups. No local anesthetic toxicity or neurological complications or falls were recorded in either group. The postoperative 24 h ROM did not differ between groups C and N. Group N had a better quality of sleep than group C (*P*=0.029). No significant differences in satisfaction scores were recorded between the two groups (Table 3).

## 4. Discussion

This clinical study is the first to assess the potential analgesic properties of FNB with a low dose of naloxone in patients undergoing TKA. We designed this trial to compare the use of 100 ng of naloxone added to ropivacaine with the known comparator ropivacaine alone and to review the effect of naloxone adjuvant in the context of FNB administration. Additionally, as the dose-responsiveness of naloxone has been validated by previous dose-range studies that investigated the optimal dose of naloxone additives [16, 17], we considered that the use of 100 ng of naloxone would represent the current standard practice. Compared with the control group, the naloxone mixed group reported postoperative pain relief, with a significant prolongation of the period before the first request for rescue analgesia. Our study also showed a decreased total opioid requirement at 24 h and good quality of sleep postoperatively. However, the onset time of the sensory blockade, satisfaction score, and complications were not significantly different between the ropivacaine alone group and the ropivacaine plus naloxone group in the setting of FNB administration.

Naloxone causes a dose-dependent pain response in a rat model. Low doses of naloxone produce paradoxical analgesia, while larger doses result in hyperalgesia [16]. Low doses of naloxone are able to displace the endogenous ligands from receptor sites or release endorphins, which has gained popularity as an opioid antagonist, and may induce an analgesic effect [16, 17, 19]. Capuano et al. [20] noted that peripheral antinociception induced by naloxone is relatively weak (approximately 40–45%) compared to that obtained with the local administration of an opioid agonist. Nevertheless, the demonstration that peripheral naloxone induces antinociception via opioidergic modulation might be of clinical interest in combined therapies where an increased analgesic effect is sought through the potentiation of peripheral mechanisms. These studies may explain the significant prolongation of the sensory block induced by a low dose of naloxone in this study. Tsai et al. [21] insisted that pretreated low-dose naloxone restores the antinociceptive effect of morphine, which is associated with a reduction in excitatory amino acid concentrations in the CSF dialysate of pertussis toxin-treated rats. There were concerns about neurotoxicity due to exposure to high concentrations of naloxone in the nerve sheath [22], but we were able to promote our research based on various previous studies described below and a study regarding expected neuroprotective effects [23].

Previous studies examining the use of naloxone to alleviate pain when added in small amounts to LA in various neural blockade procedures have been published [11–14]. Movafegh et al. [12] studied the effect of appending a low dose of naloxone to a lidocaine solution for axillary blockade and reported that this combination prolongs motor and sensory blockade, with a significantly increased interval to the first sensation of postoperative pain in the naloxone group. In addition, Al-Shukaili et al. [13] added a low dose of naloxone to bupivacaine to achieve supraclavicular brachial plexus blockade. The authors reported that naloxone increased the sensory block duration by approximately 3 h. Recovery of the sensory block was slower in the naloxone mixed group than in the control group. Similarly, Amer and Omara [14] showed that a low dose of naloxone prolonged intrapleural infiltration after surgery involving thoracotomy. Based on the previous studies, we applied it for the first time to the femoral nerve block with a low dose of naloxone to a ropivacaine solution.

The outcome of the expected effect depending on the route with low-dose naloxone is still controversial, and the mechanism and reason are not clear. Epidural naloxone was effective in reducing PONV induced by epidural sufentanil and additionally enhanced the analgesic effect [24]. In addition, Firouzian et al. [25] concluded that infusion of low-dose naloxone (0.25 mg/kg/h) along with morphine IVPCA can significantly reduce pain intensity, morphine consumption, and opioid-induced nausea and pruritus after lumbar discectomy. The results of a study by Kim et al. [26] demonstrated that the epidural administration of naloxone at a dose below 0.412 *μ*g/kg/h would be optimal for pain control and the reduction of nausea. Furthermore, intrathecal opioids combined with postoperative IV naloxone infusion provided excellent analgesia for radical prostate surgery. Moreover, IV naloxone infusion appeared to control opioid side effects without diminishing the analgesia. In addition, no serious adverse effects were noted [27]. However, Bang et al. [28] obtained the opposite results in their study. Although they administered hydromorphone on epidural, an opioid, the naloxone-treated group felt even more pain. In addition, the study conducted by Cepeda et al. [29] indicated that morphine with the naloxone group had more treatment failures, greater pain intensity, less pain relief, and higher opioid requirements than the morphine group. Moreover, the patient satisfaction was reportedly lower. The incidence of side effects was similar in both groups. In contrast to previous reports, adding low doses of naloxone to a morphine IVPCA solution increases opioid requirements and pain. Choi et al. [30] found that epidural coadministration of morphine and naloxone reduces morphine-induced side effects without reversal of the analgesic effect in patients undergoing gynecological surgery. These results are likely to have been influenced by the single or continuous administration, as the difference in a continuous infusion rate is that it maintains the continuity of the drug effect by maintaining a constant concentration of naloxone in the blood compared with a single infusion, which instantaneously increases the concentration in the blood and partially reverses the analgesic effect of opioids. Research on the addition of low-dose naloxone, which is comprehensively influenced by factors such as route and method of administration, is therefore of increasing importance.

The total dose of supplementary opioids consumed was significantly lower in group N than that in group C. Consistent with our finding, Marashi et al. [11] showed that patients in the group receiving bupivacaine alone for supraclavicular brachial plexus block consumed more morphine than patients in the naloxone groups. However, Al-Shukaili et al. [13] did not observe a difference in postoperative analgesia requirements between the naloxone-bupivacaine group and the bupivacaine alone group. A potential explanation for the difference between our findings and their results is the differences in surgical procedures used in the two studies. Overall, because our study had a limit of 24 h, the extension of the duration before the first request for analgesia within that period appears to have led to a decrease in the total dose of supplementary opioids consumed. In addition to the indirect method of observing the time and amount of pain medication required, we also recorded VAS scores, which were directly examined, although scores were recorded at a long time interval of 6 h. The pain scores at rest in both groups tended to increase over time, and group N showed a statistically significant decrease at 12 and 18 h after surgery. Group N experienced less pain during activity up to 12 h after surgery. When we combined these results, prolonged sensory blockade was observed with the additional administration of low-dose naloxone.

At up to 12 h after surgery, group C displayed greater quadricep strength than group N. The motor ability was also extended with the administration of a low dose of naloxone as an adjuvant. However, no patients experienced falls, and Tan et al. [31] reported similar results in the FNB group. The early ease of joint activities may promote the transport of intra-articular inflammatory cytokines into the blood, decrease the concentrations of inflammatory cytokines, and subsequently reduce postoperative pain and promote the further rehabilitation of patients [1, 3, 32].

Similar to the study by Marashi et al. [11], we did not show a significant difference in the onset time of sensory blockade. This result differs from the study by Movafegh et al. [12]. Additionally, because a mean difference of 5 min was observed, the result is statistically meaningful but will likely not have a significant impact clinically.

The incidence of postoperative complications, including hypotension, nausea and vomiting, pruritus, urinary retention, local anesthetic toxicity, or neurological complications, was not significantly different between the two groups. In addition, no serious side effects have been reported following both intravenous [25, 33] and intrathecal [34, 35] administration of low-dose naloxone in other studies. According to previous studies, a low dose of intravenous naloxone reduces the side effects of opioids [24, 35], but the incidence of complications in other studies was similar in all groups [28]. This discrepancy may be because our study did not use opioids or perhaps because our sample size was too small to observe any difference in the development of complications.

A significant difference in the satisfaction score, which was evaluated with a sleep-quality questionnaire administered 24 h after surgery, was not observed between the two groups. The sleep interruptions caused by pain were significantly different between the control and naloxone groups on the first night. As observed in our study, unrelieved postoperative pain may exert a significant effect on sleep. In group C, the majority of patients (41%) experienced good sleep, while the majority of patients in group N (57%) experienced a fair quality of sleep. Strassels et al. [32] reported that acute pain substantially impairs patients' sleep during the postoperative period. We did not observe acute deep periprosthetic joint infection, reoperations, or wound breakdown in either group.

The main limitations of this study are listed below. First, our results regarding secondary outcomes were only able to be generalized in the context of systemic multimodal analgesia and LIA, and the results may be different in the absence of any multimodal analgesic interventions. Second, since this is the first time that low-dose naloxone with FNB was administered, the value (258 min difference) extracted from the results of a previous study [14] was used to calculate the sample size. Each included group was relatively small and underpowered to detect significant differences that could be caused by a difference from the value (133 min difference) of this study. In the future, this study will be used as a pilot study to solve this limitation by securing a more appropriate number of samples. Third, the observations were limited to inpatients, with no long-term follow-up. Further studies that clarify the long-term outcomes of a low naloxone dose on FNB after TKA are necessary. Fourth, the observation interval was too long and wide. Changes that could occur at any moment and that sensory and motor sensations which are difficult to be checked at midpoints were not measured. However, examinations that are too frequent may cause injury and fatigue in the patients themselves and may be an obstacle to rapid recovery.

## 5. Conclusions

The addition of a low dose of naloxone to 0.375% ropivacaine for FNB prolongs the duration of the time to the first request for rescue analgesia and reduces the amount of supplementary opioids consumed without any significant adverse effects. Further studies are required to assess the most favorable dose of naloxone to be used as an adjuvant for prolonged FNB and the mechanism underlying this effect.

## Figures and Tables

**Table 1:** Patient demographics and operative data.

Variable	Group C (*n* = 37)	Group N (*n* = 37)
Age (Y)	65.3 ± 9.7	62.8 ± 6.4
Sex (male/female)	13/24	14/23
BMI (kg/m^2^)	25.7 ± 2.1	24.8 ± 2.4
Knee disease composition (RA/OA)	2/35	3/34
Surgical site (right/left)	19/18	21/16
ASA classification (1/2/3)	4/20/13	5/17/15
Preoperative VAS score (0∼10)	4.8 ± 2.8	5.0 ± 2.5
Preoperative ROM (r)	93.1 ± 7.8	94.1 ± 8.1
Operative time (min)	120.2 ± 15.9	124.8 ± 11.3
Anesthesia time (min)	185.3 ± 13.7	188.3 ± 12.3

Data are presented as the means ± SD or numbers of patients. BMI: body mass index; RA: rheumatoid arthritis; OA: osteoarthritis; ASA: American Society of Anesthesiologists; VAS: visual analog scale; ROM: range of motion; group C: control group; group N: naloxone group. No significant differences were observed between the groups.

**Table 2:** Period to the first analgesic requirement, total opioid requirement at 24 h postoperatively, and onset time of sensory blockade.

Variable	Group C (*n* = 37)	Group N (*n* = 37)	*P* value
Period to the first analgesic requirement (min)	602.6 ± 210.4	735.5 ± 187.2	0.003
Total opioid requirement at 24 h postoperatively (*μ*g)	456.5 ± 279.5	312.4 ± 141.7	0.007
Onset time of the sensory blockade (min)	22.8 ± 10.8	19.2 ± 6.3	0.084

Data are presented as the means ± SD. Group C: control group; group N: naloxone group.

**Table 3:** Complications, ROM, sleep quality, and satisfaction score following the surgery.

Variable	Group C (*n* = 37)	Group N (*n* = 37)	*P* value
Hypotension	3 (8.1)	2 (5.4)	0.999
Nausea and vomiting	6 (16.2)	5 (13.5)	0.744
Pruritus	2 (5.4)	2 (5.4)	0.999
Urinary retention	7 (18.9)	8 (21.6)	0.772
Local anesthetic toxicity	0 (0.0)	0 (0.0)	—
Neurological complications	0 (0.0)	0 (0.0)	—
Fall	0 (0.0)	0 (0.0)	—
ROM at 24 h postoperatively (°)	66.8 ± 10.4	69.1 ± 8.7	0.291
Quality of sleep (4/3/2/1/0)	12/3/1/9/12	16/5/5/9/2	0.029
Satisfaction score (0–100)	82.5 ± 4.4	83.8 ± 3.6	0.169

Data are presented as the means ± SD or numbers of patients (%). ROM: range of motion; group C: control group; group N: naloxone group.

## Data Availability

It is being stored in the hospital data server. Due to personal information issues, it cannot be provided collectively. We will provide it separately if there is a later request.

## References

[1] Capdevila X., Barthelet Y., Biboulet P., Ryckwaert Y., Rubenovitch J., d’Athis F. (1999). Effects of perioperative analgesic technique on the surgical outcome and duration of rehabilitation after major knee surgery. *Anesthesiology*.

[2] Essving P., Axelsson K., Kjellberg J., Wallgren Ö., Gupta A., Lundin A. (2010). Reduced morphine consumption and pain intensity with local infiltration analgesia (LIA) following total knee arthroplasty. *Acta Orthopaedica*.

[3] Wang H., Boctor B., Verner J. (2002). The effect of single-injection femoral nerve block on rehabilitation and length of hospital stay after total knee replacement. *Regional Anesthesia and Pain Medicine*.

[4] Abdallah F. W., Chan V. W. S., Gandhi R., Koshkin A., Abbas S., Brull R. (2014). The analgesic effects of proximal, distal, or no sciatic nerve block on posterior knee pain after total knee arthroplasty. *Anesthesiology*.

[5] Bogoch E. R., Henke M., Mackenzie T., Olschewski E., Mahomed N. N. (2002). Lumbar paravertebral nerve block in the management of pain after total hip and knee arthroplasty: a randomized controlled clinical trial. *The Journal of Arthroplasty*.

[6] Kristensen P. K., Pfeiffer-Jensen M., Storm J. O., Thillemann T. M. (2014). Local infiltration analgesia is comparable to femoral nerve block after anterior cruciate ligament reconstruction with hamstring tendon graft: a randomised controlled trial. *Knee Surgery, Sports Traumatology, Arthroscopy*.

[7] Lim Y., Quek H., Phoo W., Mah C., Tan S. (2019). A randomised controlled trial comparing adductor canal block and femoral nerve block for knee arthroplasty. *Singapore Medical Journal*.

[8] Gadsden J. C., Sata S., Bullock W. M., Kumar A. H., Grant S. A., Dooley J. R. (2020). The relative analgesic value of a femoral nerve block versus adductor canal block following total knee arthroplasty: a randomized, controlled, double-blinded study. *Korean Journal of Anesthesiology*.

[9] Crain S. M., Shen K. F. (2000). Antagonists of excitatory opioid receptor functions enhance morphine’s analgesic potency and attenuate opioid tolerance/dependence liability. *Pain*.

[10] Gan T. J., Ginsberg B., Glass P. S. A., Fortney J., Jhaveri R., Perno R. (1997). Opioid-sparing effects of a low-dose infusion of naloxone in patient-administered morphine sulfate. *Anesthesiology*.

[11] Marashi S. M., Sharifnia H. R., Azimaraghi O., Aghajani Y., Barzin G., Movafegh A. (2015). Naloxone added to bupivacaine or bupivacaine-fentanyl prolongs motor and sensory block during supraclavicular brachial plexus blockade: a randomized clinical trial. *Acta Anaesthesiologica Scandinavica*.

[12] Movafegh A., Nouralishahi B., Sadeghi M., Nabavian O. (2009). An ultra-low dose of naloxone added to lidocaine or lidocaine-fentanyl mixture prolongs axillary brachial plexus blockade. *Anesthesia & Analgesia*.

[13] Al-Shukaili A., Al-Mandhari K., Younis B. (2016). Ultra-low dose naloxone added to 0.5% bupivacaine significantly prolongs the duration of analgesia following supraclavicular brachial plexus block. *American Journal of Case Reports*.

[14] Amer A. F., Omara A. F. (2019). Small dose of naloxone as an adjuvant to bupivacaine in intrapleural infiltration after thoracotomy surgery: a prospective, controlled study. *The Korean Journal of Pain*.

[15] Bohannon R. W. (2001). Measuring knee extensor muscle strength. *American Journal of Physical Medicine & Rehabilitation*.

[16] Kayser V., Guilbaud G. (1981). Dose-dependent analgesic and hyperalgesic effects of systemic naloxone in arthritic rats. *Brain Research*.

[17] Levine J. D., Gordon N. C., Fields H. L. (1979). Naloxone dose dependently produces analgesia and hyperalgesia in postoperative pain. *Nature*.

[18] Carney C. E., Buysse D. J., Ancoli-Israel S. (2012). The consensus sleep diary: standardizing prospective sleep self-monitoring. *Sleep*.

[19] Levine J. D., Gordon N. C. (1986). Method of administration determines the effect of naloxone on pain. *Brain Research*.

[20] Capuano A., Corato A. D., Treglia M. (2010). Peripheral antinociceptive effects of low doses of naloxone in an in vivo and in vitro model of trigeminal nociception. *Neuropharmacology*.

[21] Tsai R.-Y., Jang F.-L., Tai Y.-H., Lin S.-L., Shen C.-H., Wong C.-S. (2008). Ultra-low-dose naloxone restores the antinociceptive effect of morphine and suppresses spinal neuroinflammation in PTX-treated rats. *Neuropsychopharmacology*.

[22] Kim J. P., Goldberg M. P., Choi D. W. (1987). High concentrations of naloxone attenuate N-methyl-D-aspartate receptor-mediated neurotoxicity. *European Journal of Pharmacology*.

[23] Cheng W., Li Y., Hou X. (2014). HSP60 is involved in the neuroprotective effects of naloxone. *Molecular Medicine Reports*.

[24] Kim M. K., Nam S. B., Cho M. J., Shin Y.-S. (2007). Epidural naloxone reduces postoperative nausea and vomiting in patients receiving epidural sufentanil for postoperative analgesia. *British Journal of Anaesthesia*.

[25] Firouzian A., Gholipour Baradari A., Alipour A. (2018). Ultra-low-dose naloxone as an adjuvant to patient controlled analgesia (PCA) with morphine for postoperative pain relief following lumber discectomy: a double-blind, randomized, placebo-controlled trial. *Journal of Neurosurgical Anesthesiology*.

[26] Kim E. S., Lee J., Choi J. H. (2004). Optimal dose range of epidural naloxone to reduce nausea in patients receiving epidural morphine. *Canadian Journal of Anesthesia*.

[27] Rebel A., Sloan P., Andrykowski M. (2009). Postoperative analgesia after radical prostatectomy with high-dose intrathecal morphine and intravenous naloxone: a retrospective review. *Journal of Opioid Management*.

[28] Bang S. R., Kim H. S., Kim J. H. (2006). Effects of epidural naloxone on pruritus induced by hydromorphone epidural patient-controlled analgesia. *The Korean Journal of Pain*.

[29] Cepeda M. S., Africano J. M., Manrique A. M., Fragoso W., Carr D. B. (2002). The combination of low dose of naloxone and morphine in PCA does not decrease opioid requirements in the postoperative period. *Pain*.

[30] Choi J. H., Lee J., Choi J. H., Bishop M. J. (2000). Epidural naloxone reduces pruritus and nausea without affecting analgesia by epidural morphine in bupivacaine. *Canadian Journal of Anesthesia*.

[31] Tan Z., Kang P., Pei F. (2018). A comparison of adductor canal block and femoral nerve block after total-knee arthroplasty regarding analgesic effect, effectiveness of early rehabilitation, and lateral knee pain relief in the early stage. *Medicine (Baltimore)*.

[32] Strassels S. A., McNicol E., Wagner A. K., Rogers W. H., Gouveia W. A., Carr D. B. (2004). Persistent postoperative pain, health-related quality of life, and functioning 1 month after hospital discharge. *Acute Pain*.

[33] Sartain J. B., Barry J. J., Richardson C. A., Branagan H. C. (2003). Effect of combining naloxone and morphine for intravenous patient-controlled analgesia. *Anesthesiology*.

[34] Block L., Lundborg C., Bjersing J., Dahm P., Hansson E., Biber B. (2015). Ultralow dose of naloxone as an adjuvant to intrathecal morphine infusion improves perceived quality of sleep but fails to alter persistent pain. *The Clinical Journal of Pain*.

[35] Firouzian A., Gholipour Baradari A., Ehteshami S. (2020). The effect of ultra-low-dose intrathecal naloxone on pain intensity after lumbar laminectomy with spinal fusion: a randomized controlled trial. *Journal of Neurosurgical Anesthesiology*.

